# Effect of Strain, Wood Substrate and Cold Treatment on the Yield and *β*-Glucan Content of *Ganoderma lucidum* Fruiting Bodies

**DOI:** 10.3390/molecules25204732

**Published:** 2020-10-15

**Authors:** Marta Cortina-Escribano, Juha-Matti Pihlava, Jari Miina, Pyry Veteli, Riikka Linnakoski, Henri Vanhanen

**Affiliations:** 1 School of Forest Sciences, University of Eastern Finland, 80100 Joensuu, North Karelia, Finland; 2 Natural Resources Institute Finland (Luke), 80100 Joensuu, North Karelia, Finland; jari.miina@luke.fi (J.M.); henri.vanhanen@luke.fi (H.V.); 3 Natural Resources Institute Finland (Luke), 31600 Tavastia Proper, Jokioinen, Finland; juha-matti.pihlava@luke.fi; 4 Natural Resources Institute Finland (Luke), 00790 Uusimaa, Helsinki, Finland; pyry.veteli@gmail.com (P.V.); riikka.linnakoski@luke.fi (R.L.)

**Keywords:** Bioconversion, boreal, circular economy, *Ganoderma lucidum*, mushroom cultivation, polysaccharides, wood side stream, *β*-glucan

## Abstract

Wood residues from forestry industries can be potential raw materials for specialty and edible mushroom production. The aim of this study was to evaluate the suitability of wood residues for the cultivation of *Ganoderma lucidum* originating from boreal forests. The substrates tested included sawdust and wood chips of *Betula* spp., *Populus tremula*, *Picea abies*, *Pinus sylvestris* and *Larix* sp. The suitability of the substrates and the ability of the strains to develop fruiting bodies and produce *β*-glucan were evaluated. Fruiting body formation was supported by applying two different cold shock treatments to substrate bags. The highest yields were observed with MUS192 strain and *Betula* spp. and *P. tremula* wood-based substrates. *β*-Glucan content in the fruiting bodies was highest with the MUS75 and *P. tremula* wood-based substrate. Based on these findings, the combination of *P. tremula* wood residues and the MUS192 strain is proposed to enhance the yield and *β*-glucan content of the fruiting bodies. A cold treatment of 5 °C is suggested to induce primordia formation and to increase fruiting probability. This is the first time that strains of *G. lucidum* originating from boreal forests have been compared and successfully cultivated simulating commercial indoor cultivation.

## 1. Introduction

High volumes of wood-based side streams are generated from wood processing industries in Europe. In Finland, sawmill and plywood industries alone produced in 2016 over 1.2 million cubic meters of side streams [1]. One of the threats associated with the utilization of wood-based side streams is the scarce development of new products and the expensiveness of the process [2]. Wood-based side streams, mainly composed of wood chips, sawdust and bark, are mostly used for energy and pulp production in Northern Europe [2]. The main tree species grown and used in sawmill and plywood industries in Finland are conifer species (*Pinus sylvestris* and *Picea abies*) and birch (*Betula pendula* and *Betula pubescens*) [1]. Other species available [3] are used on a minor scale such as *Populus tremula* and *Larix* sp. The high availability of these resources along with new European policies towards a circular economy [4] have pushed initiatives to find new uses for these side streams. Moreover, wood processing industries are seeking the optimization and revalorization of their resources.

Wood side streams have been found to be a potential raw material for specialty and edible mushroom production [5]. Mushroom cultivation allows the inexpensive transformation of lignocellulosic biomass into high-value products such as polysaccharides. Moreover, the biotechnological process is supported by the large availability and accessibility of the substrate material. In mushroom cultivation, the production of fruiting bodies requires long periods and the quality of the products is affected by the environmental conditions and substrate. Hence, it is very important to select the most suitable strains, growing substrate and environmental conditions for the commercial cultivation of mushrooms. With an adequate cultivation technique, agroforestry and agroindustrial by-products can be recovered for the production of edible and specialty mushrooms. Some of these by-products include sawdust, tea waste, wheat straw, cotton waste, peanut shells, soy residues and olive mill wastewaters, among others [6,7,8,9,10,11].

Among cultivated fungal species, *Ganoderma* is a popular genus including pathogenic and wood-degrading species that have been studied in regard to their bioactivity [12]. The most extensively produced and economically important specialty mushroom in China (from where its cultivation practices originate) is *Ganoderma lucidum* [13]. *G. lucidum* has been cultivated in a wide variety of substrates including sawdust from *Acacia* sp., *Alnus* sp., *Carpinus* sp., *Dalbergia* sp., *Dipterocarpus* sp., *Fagus* sp., *Pinus* sp., *Populus* sp., *Quercus* sp. and *Swietenia* sp., among others [6,7,8,14,15,16,17]. *Ganoderma* contains several compounds that define the bioactive and pharmacological properties of the fungus such as antiviral [18], immunomodulatory, antitumor, antioxidant, hepatoprotective or antihypertensive activities [19]. Polysaccharides, along with triterpenoids, are the major bioactive constituents of the *Ganoderma* genus [12]. Among them, *β*-glucan is a dominant structural polysaccharide that defines the shape and rigidity of the cell, serving as a reservoir of carbohydrates [20]. This polysaccharide is mainly found in the fungal cell walls, but it can be found inside the cell or secreted in the medium where the fungus grows [21]. Due to its bioactive properties, *β*-glucan has numerous applications in the pharmaceutical and food industries [20]. To supply its demand, attempts to enhance the production of fungal polysaccharides by modifying and optimizing the cultivation conditions and growing media have been carried out [10,16,22,23,24,25,26,27].

In Asian countries, ‘*Ganoderma lucidum*’ has been used to name the traditionally cultivated Reishi or Ling-Zhi mushroom [13]. However, the taxonomy of the species is not clear leading to confusion among closely related species [28,29,30]. The genus *Ganoderma* (P. Karst.) was described by Karsten [31], combining only the species type *G. lucidum* (W. Curtis) Karst. into the new genus. Curtis, the author of the epitheton, based his description on a specimen from the UK [32]. Since then, the fungus has been reported worldwide based mainly on morphological characteristics [33,34]. Recently, molecular and taxonomic data have confirmed that Asian ‘*G. lucidum*’ differs from the originally described species from Europe [28,29,30].

*Ganoderma lucidum* is naturally distributed in Finnish boreal forests. Its distribution is sporadic and it is commonly found in coastal areas [35], but the species can also be found inland; the northernmost findings are above the Arctic Circle [36]. In Finland, *G. lucidum* has been found in stumps of *Alnus glutinosa*, *Betula* sp., *P. abies*, *Quercus robur* and *P. sylvestris* [35]. Currently, the cultivation of *G. lucidum* in Finland is still in its infancy. An attempt to cultivate Finnish strains of *G. lucidum* in vitro using wood residues has been carried [37]. However, the suitability of wood side streams for the production of *G. lucidum* fruiting bodies and *β*-glucan using strains originating from boreal forests has not been investigated.

In this work, different wood side streams of sawmill and plywood industries were tested as substrate media for the cultivation of *G. lucidum* strains originating from boreal forests. The aim is to find the most suitable strain, wood substrate and cold treatment for the production of *G. lucidum* fruiting bodies and *β*-glucan. The strain selection and cold shock treatment were critical for successful fruiting. The wood substrate had the strongest effect on the yield and *β*-glucan content of the fruiting bodies. *Betula* spp. and *Populus tremula* wood side streams were found the most suitable substrates to enhance the yield of *G. lucidum* fruiting bodies. The highest content of *β*-glucan was obtained in the fruiting bodies growing in the *P. tremula* wood-based substrate.

## 2. Results

### 2.1. Effect of Strain, Wood Substrate and Cold Treatment on Successful Fruiting

The suitability of strains (MUS192, MUS6, MUS75, MUS9, MUS12 and MUS19), wood substrates (*Betula* spp., *P. tremula*, *P. abies*, *P. sylvestris* and *Larix* sp.) and cold treatments (5 °C and −20 °C) for the cultivation of *G. lucidum* were evaluated by their capability of producing fruiting bodies. From the total substrate bags (N = 300), only 31.7% the produced fruiting bodies (N = 95), of which 89.5% were exposed to 5 °C (N = 85) and 10.5% to −20 °C (N = 10). Substrate bags exposed to −20 °C did not produce fruiting bodies and were susceptible to contamination. Successful fruiting bags exposed to −20 °C were inoculated with the MUS19 strain into *P. tremula* and *P. abies* wood-based substrates.

From a total of 50 bags per strain, strains MUS19, MUS192 and MUS6 were more capable of producing fruiting bodies (N = 30, 20 and 18, respectively) than MUS9, MUS12 and MUS75 (N = 14, 9 and 4, respectively). Regarding the wood substrates (60 bags per substrate), *P. tremula* and *Betula* spp. were found more suitable for fruiting (N = 27 and 20, respectively) than *P. abies*, *P. sylvestris* and *Larix* sp. (N = 18, 17 and 13, respectively). Substrate colonization was completed within one month for most of the wood substrates except for *P. sylvestris* and *Larix* sp. wood-based substrates, in which colonization was slow or uncompleted.

A logistic regression model (Equation (1)) was fitted to predict the probability of the successful fruiting of *G. lucidum* as a function of strains, wood substrates and cold treatments (Table 1). Treatment significantly predicted the probability of fruiting (Wald χ^2^ = 58.78, *p* < 0.001) indicating that cold treatment in +5 °C increased the probability of a successful fruiting body in comparison to treatment at −20 °C. The fungal strain (Wald χ^2^ = 41.82, *p* < 0.001), followed by wood substrate (Wald χ^2^ = 13.97, *p* < 0.001), significantly predicted the probability of the fruiting of *G. lucidum*. For instance, the inoculation of MUS75 or MUS12 would significantly decrease the probability of fruiting (using MUS19 as reference). On the contrary, the utilization of the wood substrate based on *P. tremula* would significantly increase the probability of fruiting (using *P. abies* as a reference).

### 2.2. Effect of Strain, Wood Substrate and Cold Treatment on Yield

The effect of strain, wood, treatment and their interactions on the fruiting body yield of *G. lucidum* was studied using the substrate bags that produced fruiting bodies. The linear regression model expressed in Equation (2) showed that the effect of strain and wood had a significant effect (*p* < 0.05) on the yield of the fruiting bodies of *G. lucidum* (Table 2). Wood substrate had the strongest effect on the yield (F (4,69) = 9.71)). The effect of cold treatment and the interaction between the main factors did not show a significant effect on the yield (*p* > 0.05). For example, the interaction of wood and strain was not significant (F (14,69) = 1.12; *p* = 0.361).

**Figure 1:**
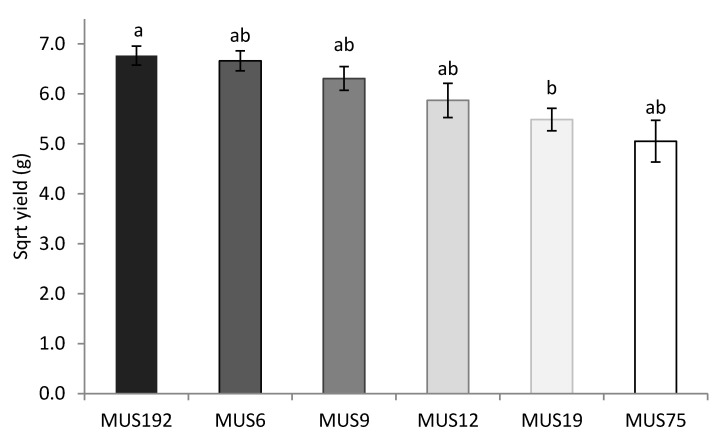
Effect of strain on the fruiting body yield of *G. lucidum* estimated in Equation (2). Significant differences (*p* < 0.05) between the estimated square root yields are represented with different letters (a and b).

The effect of strain on the fruiting body yield is presented in more detail in Figure 1. The only significant differences on the estimated yield were observed between the strains MUS192 and MUS19 (*p* < 0.05). These strains presented fruiting bodies with the highest probability of success fruiting bodies in comparison with other strains (Table 1). The yields per bag were 51.3 g and 37.2 g, respectively, for the MUS192 and MUS19 strains.

**Figure 2:**
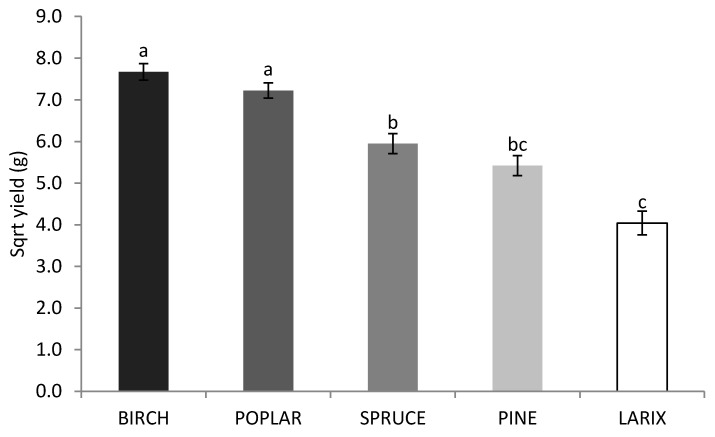
Effect of the wood substrate on the fruiting body yield of *G. lucidum* estimated in Equation (2). Significant differences (*p* < 0.05) between the square root yields are represented with different letters (a, b and c).

In Figure 2, the effect of the wood substrate on the yield of the fruiting body of *G. lucidum* is shown. The highest estimated yields were obtained with the *Betula* spp. and *P. tremula* wood-based substrate (not statistically different from each other). The yields per bag were 59.8 g and 52.1 g, respectively, for *Betula* spp. and *P. tremula*. However, the highest yield per bag (90.2 g) was observed in the *P. tremula* wood-based substrate inoculated with the MUS192 strain. The *P. abies* and *P. sylvestris* wood-based substrates were not as suitable as deciduous species for the yield of fruiting bodies. *Larix* sp. was found the least suitable for the yield of *G. lucidum* fruiting bodies (not statistically different from *P. sylvestris*).

### 2.3. Effect of Strain and Wood Substrate on Glucan Contents

The effect of strain, wood and their interactions on glucan contents of *G. lucidum* fruiting bodies was studied using the substrate bags treated at +5 °C (Equation (3)). As expected, the strain and wood substrate had a significant effect (*p* < 0.05) on the contents of total glucan and *β*-glucan in the fruiting bodies of *G. lucidum* (Table 3). On the contrary, the factors did not have an effect on the content of *α*-glucan. Wood had the highest effect on the total glucan and *β*-glucan content in fruiting bodies (F = 17.28 and F = 15.60, respectively). Similarly to the results of fruiting body yield (Table 2), the interaction between the main factors strain and wood did not have a significant effect on the production of glucans.

**Figure 3:**
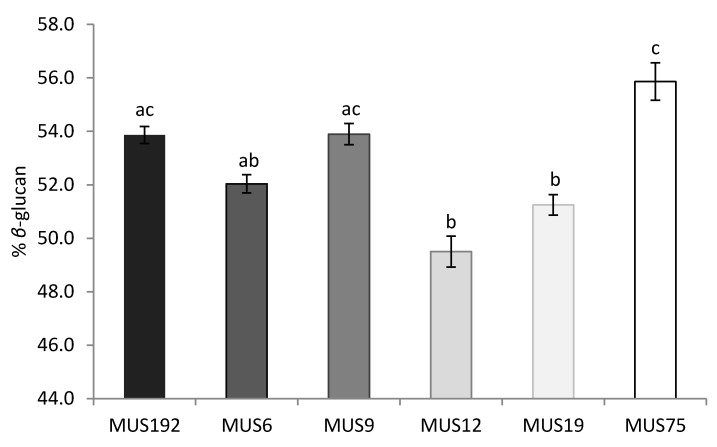
Effect of strain on the *β*-glucan content of *G. lucidum* fruiting bodies estimated in Equation (3). Significant differences (*p* < 0.05) between the square root yields are represented with different letters (a, b and c).

In Figure 3, the effect of strains on the *β*-glucan content of the fruiting bodies of *G. lucidum* is represented in detail. The MUS75 strain presented the highest *β*-glucan content (marginal mean of 55.9% of *β*-glucan; not statistically different from MUS192 and MUS9) even though it was the strain with the lowest probability of fruiting (Table 1) and fruiting body yield (Figure 1). MUS12 and MUS19 presented the lowest *β*-glucan content (49.5 and 51.2%, respectively; not statistically different from MUS6).

**Figure 4:**
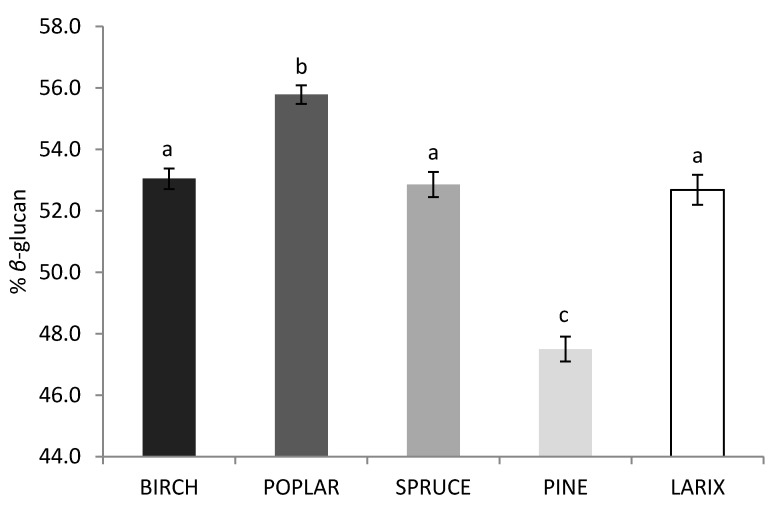
Effect of the wood substrate on the *β*-glucan content of *G. lucidum* fruiting bodies estimated in Equation (3). Significant differences (*p* < 0.05) between the square root yields are represented with different letters (a, b and c).

The *β*-glucan content of the fruiting bodies of *G. lucidum* was affected by the tree species used as wood substrate (Figure 4). The highest *β*-glucan content was estimated for the *P. tremula* wood-based substrate (marginal mean of 55.8% of *β*-glucan). The *P. tremula* wood-based substrate also presented a significantly higher probability of fruiting (Table 1) and fruiting body yield (not statistically different from *Betula* spp.; Figure 2) in comparison with the other species. The *P. sylvestris* wood-based substrate significantly presented the lowest content of *β*-glucan in the fruiting bodies (marginal mean of 47.5% of *β*-glucan).

## 3. Discussion

The effect of strain, wood substrate and cold treatment on the probability of successful fruiting, yield and *β*-glucan content of the fruiting bodies of *G. lucidum* was studied in this work. The strain selection and the cold shock treatment had the strongest effects on the probability of fruiting. The wood substrate had the strongest effect on the yield and *β*-glucan content in the fruiting bodies. The highest yields were obtained with *Betula* spp. and *P. tremula* wood side streams. *P. tremula* was found to be the most suitable substrate to enhance the *β*-glucan content in the fruiting bodies.

Cold treatment was needed after full colonization of the substrate bags to induce primordia formation. In this study, the cold treatment was adequate at 5 °C. When a lower temperature (−20 °C) was applied, the substrate bags did not produce fruiting bodies and were susceptible to contamination. In a previous study, a cold treatment at 5 °C decreased the time needed for fruiting and enhanced the biological efficiency of *Pleurotus* sp. [38]. Fungal strains belonging to the same species may behave differently to temperature depending on the climate conditions of their origin [39]. Due to the boreal climate conditions of the strains’ origin, it is possible that environmental stress (i.e., cold shock) is required to trigger the formation of fruiting bodies.

Previously, the effect of wood sawdust from the same tree species on the mycelial growth of *G. lucidum* was tested in agar media [37]. In accordance with the results presented in the current study, *Betula* spp. and *P. tremula* sawdust-amended media enhanced the mycelial growth of *G. lucidum* in vitro. Therefore, it is not surprising to observe a higher probability of fruiting using wood residues of those tree species as substrate.

The yield was found also higher in *Betula* spp. and *P. tremula* in comparison with conifer species. The interaction of strain and wood did not have a significant effect on the probability of fruiting, yield or *β*-glucan production. Surprisingly, the maximum yield and biological efficiency of 90.2 g and 9% were achieved with the poplar wood-based substrate. In Finland, *G. lucidum* has not been reported growing in *P. tremula* in nature [35]. Higher biological efficiencies, 22.6% (alder sawdust supplemented with gram flour) and 20.7% (oak sawdust supplemented with corn bran), respectively, were obtained by Gurung et al. [7] and Erkel [14]. Biological efficiency is commonly used to determine the efficiency of the substrate and strain combination to produce fruiting bodies. It is possible to increase biological efficiency using a smaller volume (<1 kg dry weight) of the wood-based substrate resulting in a similar fruiting body yield. For instance, Azizi et al. [17] reported a similar yield (80.4 g/kg) using a poplar sawdust substrate but higher biological efficiency (12.9%) in comparison with this study. Supplements to the substrate might increase the yield of *Ganoderma* fruiting bodies [6,7,14,17].

In this study, *G. lucidum* fruiting bodies had a very high content of *β*-glucan (47.5–55.9 g/100 g dm). The high content of *β*-glucan indicates that *G. lucidum* fruiting bodies may be a source for bioactive compounds for the food and pharmaceutical industries. Similar levels were obtained by McClearly and Draga [40] using the same Megazyme assay (54–54.8 g/100 g dm). The fruiting bodies analyzed presented a *β*-glucan content as high as other fungal species such as *Trametes versicolor* or *Piptoporus betulinus* (60.8 and 51.8 g/100 g dm, respectively) [26]. However, Cho et al. [41] reported a lower *β*-glucan content (<20 g/100 g dm) for *G. lucidum* specimens originating from Korea, Japan, Taiwan, USA and Brazil. Considering the taxonomy confusion of *G. lucidum*, it is possible that these specimens differ from the European *G. lucidum* [30]; therefore, differences in *β*-glucan production could be expected between closely related species. Another possible explanation is the extraction method. For instance, Benito-Román et al. [42] tested different extraction conditions and noted that the temperature and solvent volume played a critical role in the content of *β*-glucan (range between 24.0 and 62.2%) in *G. lucidum* using pressurized hot water as a solvent. The material analyzed may have an effect on the *β*-glucan content. The fruiting bodies analyzed in this study consisted of a mixture of cap and stipe. A higher content of *β*-glucan was detected in the stipe than in the cap of several wild mushrooms [26] and cultivated fungi, i.e., *Lentinula edodes* [43]. Differences of *β*-glucan content within the fruiting body remain to be studied in *G. lucidum*.

The synthesis of *β*-glucan is catalyzed by *β*-1,3-glucan synthase [21]. *β*-1,3-glucan synthase activity in *L. edodes* has been hypothesized to be associated with the presence of phenolic compounds in the media [10]. The presence of phenolic compounds in the substrate generates a stressful oxidative environment for some fungi [44]. In response to the stressful event, Reverberi et al. [10] suggested that antioxidative enzymes such as *β*-glucan synthases are activated in order to preserve the integrity of the cell wall. Therefore, *β*-glucan synthesis could be related to the defense mechanism of the cell wall [21].

Generally, conifer species contain higher levels of phenolic compounds than deciduous species; therefore, it would be expected that fruiting bodies grown in a conifer wood-based substrate presented higher *β*-glucan content. However, in this study, *β*-glucan content in the fruiting bodies was higher in the *P. tremula* wood-based substrate than in other species, especially when compared to the *P. sylvestris* wood-based substrate. Similar results were obtained by Kuhar et al. [16]. Moreover, they compared the extracts of poplar and pine wood-based substrates prior to and after mushroom cultivation; poplar contained a higher level of alkaline extract in both scenarios [16]. Alkaline extract was associated with the presence of phenolic compounds in the substrate. Considering that the nature of phenolics in deciduous species differs from conifer species, their composition and structure could be expected to have a different effect on fungal metabolism [45]. Since *P. tremula* is not a typical host species for *G. lucidum* in Finland [35], it is possible that wood substrate based on this species causes stress to *G. lucidum*. Other wood components such as lignin, cellulose and hemicellulose fractions [9,27], fatty acids [22] and resin acids [11,46], among others, could also play a role in fungal polysaccharide production. For instance, the presence of hemicellulose promoted the production of polysaccharides in the mycelia of *L. edodes* [27]. Variability in polysaccharide content in *G. lucidum* fruiting bodies and mycelia can be also due to differences pH levels and C:N ratio, carbon, nitrogen, phosphate and magnesium source of the media [23,24,25].

*Ganoderma lucidum* originating from boreal forest was successfully cultivated using wood side streams of plywood and sawmill industries. Cold shock treatment was required to trigger fruiting body formation. The *P. tremula* wood-based substrate enhanced *β*-glucan content in the fruiting bodies. The availability of wood residues and the potential of *G. lucidum* strains to produce fruiting bodies offer an opportunity to increase the valorization of wood side streams and to produce high valuable mushrooms and compounds such as *β*-glucan. This is the first study to simulate commercial indoor cultivation of *G. lucidum* strains originating from boreal forests.

## 4. Materials and Methods

### 4.1. Fungal Strains

Fungal strains were isolated in our previous study from wild fruiting bodies of *G. lucidum* and identified based on the internal transcribed spacer (ITS) DNA region sequence data [37]. The specimens were collected from wooden stumps of *P. abies* and *B. pubescens* located in the Satakunta and Uusimaa regions, Finland (Table 4). The strains are preserved in the Fungal Culture Collection of Natural Resources Institute Finland (Luke), Helsinki. The cultures were maintained at 25 °C in 2% potato dextrose agar (PDA: 20 g potato dextrose agar/L (MP Biomedicals, LLC, Illkrich, France)) and subcultured once a month.

### 4.2. Spawn Production

Common barley was used as a spawn medium. Barley grains were boiled for 2–3 h and drained to prevent excessive moisture. Preboiled barley was introduced in polypropylene bags of 5 L and autoclaved for 45 min at 125 °C and 1.2 bar. After cooling, 2 mm^2^ agar sections of the actively growing margin of the mycelium were transferred from PDA plates to the sterilized polypropylene bag. Spawn bags were sealed, shaken and kept at 22 °C in dark conditions until full colonization.

### 4.3. Substrate Preparation

Wood residues from *Betula* spp., *P. tremula*, *P. abies*, *P. sylvestris* and *Larix* sp. were used as substrate. The *Betula* spp. substrate consisted of a mixture of *B. pubescens* and *B. pendula* wood residues obtained from a local plywood company (Joensuu, North Karelia, Finland). Wood residues from *P. tremula*, *P. abies* and *P. sylvestris* and *Larix* sp. were obtained from small sawmills companies located in North Karelia and South Savo, Finland. Polypropylene bags were filled with a mixture of wood chips (particle size from 20 to 50 mm) and sawdust (ca. 0.8 mm particle size). Dry weight bags (60% wood chips and 40% sawdust by weight) of 1 kg were prepared with *P. tremula*, *P. abies*, *P. sylvestris* and *Larix* sp. The substrate bags containing *Betula* spp. were filled entirely with wood chips (particle size from 20 to 50 mm). Ten replicates of 1 kg (dry weight) per each combination of wood type and strain were prepared. Wood moisture content on a dry basis [47] was adjusted to 65–75%.

Substrate bags were autoclaved for 45 min at 125 °C and 1.2 bar. After cooling down, the substrate bags were inoculated with 0.3 L of fully colonized spawn. Substrate bags were sealed, shaken and kept at 22 °C in dark conditions until full colonization. After full colonization, substrate bags were transferred to a cropping greenhouse with a relative humidity of 65 to 75% and light exposure (8/24 h) to induce fruiting body formation.

### 4.4. Cold Treatment and Fruiting Body Yield

After several weeks, there was no observation of primordia formation. Because of the difficulties of strains to produce fruiting bodies, substrate bags were submitted to two different cold shock treatments. Half of the replicates were kept at 5 °C and the other half at −20 °C for three months. After the cold treatment, the substrate bags were maintained at 25 °C and a relative humidity of 90% until fruiting body formation. Fruiting bodies were harvested, and the yield was determined based on fresh weight (g). Harvested fruiting bodies were kept at −20 °C.

### 4.5. β-Glucan Content Analysis

The fruiting bodies were freeze-dried (Nature Lyotech Ltd., Finland) and kept at −20 °C. Fruiting bodies including both cap and stipe were cut into pieces and then milled with a hammer mill with a 1 mm sieve (KT-120, Koneteollisuus Oy, Klaukkala, Finland). Total glucan and *α*-glucan content were measured for each individual fruiting body according to McCleary and Draga [40] and using the Yeast and Mushroom *β*-glucan assay kit. In brief, the principle of the assay is to solubilize and hydrolyze the total glucan of the sample using sulfuric acid, exo-1, 3-*β* glucanase and *β*-glucosidase enzymes. *α*-Glucan and sucrose were dissolved using sodium hydroxide. Glucose was measured with amyloglucosidase and invertase using GOPOD reagent. *β*-Glucan was determined by subtracting *α*-glucan from the total glucan measurements. Total glucan, *α*-glucan and *β*-glucan content are expressed as a percentage of the freeze-dried fruiting body (g/100 g of the dry material). Analyses were done as duplicates; two samples from each pulverized fruiting body were measured separately.

### 4.6. Statistical Analysis

The effects of *G. lucidum* strain, wood substrate and cold treatment on successful fruiting (0/1), yield (g of the fresh fruiting body) and total glucan, *α*-glucan and *β*-glucan content (% of freeze-dried material) were evaluated by regression modeling. To avoid overfitting, only the two-way interactions of strain, wood and treatment were considered during modeling.

A logistic regression model was fitted for the probability of successful fruiting (Equation (1)) and a linear regression model was fitted for the yield of fruiting bodies, if any (Equation (2)):

(1) $$log\left(\frac{p_{ijlk}}{1-p_{ijlk}}\right)=S_{j}+W_{l}+Tr_{k}+S_{j}*W_{l}+S_{j}*Tr_{k}+W_{l}*Tr_{k}$$

(2) $$sqrt\left(y_{ijlk}\right)=S_{j}+W_{l}+Tr_{k}+S_{j}*W_{l}+S_{j}*Tr_{k}+W_{l}*Tr_{k}+e_{ijlk}$$

where *p* and *y* are, respectively, the probability of successful fruiting and the fruiting body yield (g of the fresh fruiting body) of the substrate bag (*i* = 1, 2, …, 5), which produced fruiting bodies; *S* is the strain of *G. lucidum* (*j* = 1, 2, …, 6); *W* is the wood substrate (*l* = 1, 2, …, 5); *Tr* is cold treatment (*k* = 1, 2); and *e* is the random error term with a mean of 0 and constant variance. Square root transformation was needed to meet assumptions for linear regression (i.e., normality and constant variance of random error terms).

The content of glucan was modeled by fitting a linear mixed model (Equation (3)):

(3) $$\%Glucan_{i’ijlk}=S_{j}+W_{l}+Tr_{k}+S_{j}*W_{l}+S_{j}*Tr_{k}+W_{l}*Tr_{k}+u_{ijlk}+e_{i^{\prime }ijlk}$$

where %*Glucan* is the content of total, *α* or *β*-glucan (% of freeze-dried material and *u* is the random substrate bag effect with a mean of 0 and constant variance. A random fruiting body effect was included in the model due to two individual samples (*i*′ = 1, 2) of each fruiting body.

The models were fitted and the marginal means were estimated for all level combinations of the factors (strains, wood substrates and cold treatments) using the MIXED procedure in IBM SPSS Statistics 25 (IBM SPSS Inc., Chicago, IL, USA). If the model involved random effects (*u*), the predicted means were computed by averaging the random effects over subjects (i.e., fruiting bodies). Using a given cold treatment, all pairwise comparisons of strain and wood substrates were performed to find out which strains and wood substrates have a statistically significant higher yield and glucan content.

## Figures and Tables

**Table 1:** The parameter estimates, their standard errors (SE) and Wald χ^2^ tests for the terms in the logistic regression model (Equation (1)) for the probability of the successful fruiting of *G. lucidum*. *p*-values are calculated for the variables in the model based on Wald’s chi-square statistics.

Variables	Estimate	SE	Wald χ^2^	*p*-Value
Constant	−1.30	0.57	5.20	0.023
Strain (df = 5, ref. MU19)			41.82	<0.001
MUS192	−1.81	0.65	7.74	0.005
MUS6	−2.15	0.66	10.54	0.001
MUS75	−4.82	0.82	34.52	<0.001
MUS9	−2.80	0.68	16.86	<0.001
MUS12	−3.65	0.72	25.89	<0.001
Wood (df = 4, ref. *P. abies*)			13.97	0.007
*Betula* spp.	0.30	0.55	0.30	0.583
*Larix* sp.	−0.81	0.58	1.98	0.160
*P. sylvestris*	−0.15	0.56	0.08	0.781
*P. tremula*	1.33	0.56	5.61	0.018
Treatment +5 °C (df = 1, ref. −20 °C)	4.14	0.54	58.78	<0.001

**Table 2:** Effect of the factors strain, wood and treatment on the yield of *G. lucidum* (Equation (2), Type III test).

Source	Numerator df	Denominator df	F	*p*-Value
Intercept	1	69	308.99	<0.001
Strain	5	69	3.40	0.008
Wood	4	69	9.71	<0.001
Treatment	1	69	0.48	0.492
Strain * Wood	14	69	1.12	0.361
Wood * Treatment	1	69	0.15	0.697

Note: The interaction between variables is shown with an asterisk between variables names.

**Table 3:** Effect of the factors strain and wood on the total glucan, *α*-glucan and *β*-glucan content of *G. lucidum* fruiting bodies of the substrate bags treated at +5 °C (Equation (3), Type III test).

Source	Num. Df	Den. Df	Total Glucan		*α*-Glucan		*β*-Glucan	
			F	*p*-Value	F	*p*-Value	F	*p*-Value
Intercept	1	61	23,708.09	<0.001	204.55	<0.001	19,374.09	<0.001
Strain	5	61	3.63	0.006	1.11	0.364	3.31	0.010
Wood	4	61	17.28	<0.001	1.37	0.256	15.60	<0.001
Strain * Wood	14	61	1.15	0.335	1.14	0.348	1.09	0.385

Note: The interaction between variables is shown with an asterisk between variables names.

**Table 4:** *Ganoderma lucidum* strains.

Strain	Region	Host Species	Isolation Date	ITS GenBank ID
MUS192	Uusimaa	*P. abies*	07/2016	MT334582
MUS6	Uusimaa	*P. abies*	07/2016	MT334583
MUS75	Uusimaa	*P. abies*	07/2016	MT334584
MUS9	Uusimaa	*P. abies*	07/2016	MT334585
MUS12	Satakunta	*P. abies*	10/2016	MT334586
MUS19	Uusimaa	*B. pubescens*	03/2017	MT334587
